# A novel high-affinity potassium transporter SeHKT1;2 from halophyte *Salicornia europaea* shows strong selectivity for Na^+^ rather than K^+^

**DOI:** 10.3389/fpls.2023.1104070

**Published:** 2023-02-20

**Authors:** Yakupjan Haxim, Lei Wang, Zhendong Pan, Xiaorong Fan, Jinbiao Ma

**Affiliations:** ^1^ State Key Laboratory of Desert and Oasis Ecology, Key Laboratory of Ecological Safety and Sustainable Development in Arid Lands, Xinjiang Institute of Ecology and Geography, Chinese Academy of Sciences, Ürümqi, China; ^2^ Xinjiang Key Laboratory of Conservation and Utilization of Plant Gene Resources, Xinjiang Institute of Ecology and Geography, Chinese Academy of Sciences, Ürümqi, China; ^3^ Turpan Eremophytes Botanical Garden, Chinese Academy of Sciences, Turpan, China; ^4^ State Key Laboratory of Crop Genetics and Germplasm Enhancement, MOA Key Laboratory of Plant Nutrition and Fertilization in Low-Middle Reaches of the Yangtze River, Nanjing Agricultural University, Nanjing, China

**Keywords:** *Salicornia europaea*, salt tolerance, high-affinity K^+^ transporters, HKT, ion selectivity

## Abstract

High-affinity K^+^ transporters (HKTs) are known as transmembrane cation transporters and are involved in Na^+^ or Na^+^-K^+^ transport in plants. In this study, a novel HKT gene *SeHKT1;2* was isolated and characterized from the halophyte, *Salicornia europaea*. It belongs to subfamily I of HKT and shows high homology with other halophyte HKT proteins. Functional characterization of *SeHKT1;2* indicated that it contributes to facilitating Na^+^ uptake in Na^+^-sensitive yeast strains G19, however, cannot rescue the K^+^ uptake-defective phenotype of yeast strain CY162, demonstrating SeHKT1;2 selectively transports Na^+^ rather than K^+^. The addition of K^+^ along with NaCl relieved the Na^+^ sensitivity. Furthermore, heterologous expression of *SeHKT1;2* in *sos1* mutant of *Arabidopsis thaliana* increased salt sensitivity and could not rescued the transgenic plants. This study will provide valuable gene resources for improving the salt tolerance in other crops by genetic engineering.

## Introduction

Globally, soil salinity remains a significant abiotic stress for plants and constraints agriculture development. In order to solve the current problems of salt stress, we must understand how salt stress tolerance works and develop salt-tolerant crops. In addition to osmotic stress, high salinity can cause plant death and growth inhibition. A primary consequence of ionic stress is sodium disequilibrium in many plants, especially gramineous crops, which can adversely affect plant nutrition, enzyme activity, photosynthesis, and metabolism (Tester and Davenport, 2003; Mahajan and Tuteja, 2005). Na^+^ is attracted to the plasma membrane in saline environments due to high external Na^+^. A number of mechanisms are involved in plants acquiring salt tolerance, including the outright exclusion of Na^+^ and vacuolar compartmentalization. Na^+^ is extruded out of salt-stressed cells by plasma membrane Na^+^/H^+^ antiporters, also called SOS1 or NHA-type transporters. NHX-type antiporters, also known as Na^+^/H^+^ antiporters, sequestrate Na^+^ from the cytosol into vacuoles when salt stress is applied to the cell (Hasegawa et al., 2003; Zhu, 2003; Mahajan et al., 2008). In addition to controlling Na^+^ influx, salt tolerance can also be improved by reducing the amount of sodium absorbed. A number of studies have indicated that toxic Na^+^ influx into roots is mediated by nonselective cation channels (NSC) or voltage-independent channels (VIC) under high external Na^+^ concentrations (Amtmann and Sanders, 1998; Schachtman and Liu, 1999; Blumwald et al., 2000). In spite of this, NSC/VIC remains mysterious in terms of their detailed molecular identities. As a result, plant cells may be able to uptake Na^+^ through high-affinity K^+^ transporters (HKT-type transporters).

Based on amino acid in the first pore domain, the HKT transporters can be classified into two subclasses. Class I HKT transporters possess a serine (S) whereas members of class II have a glycine (G) residue at first pore forming region (Mäser et al., 2002b; Almeida et al., 2013). A single amino acid at this position determines the ion selectivity of the transporter. The class I HKT transporters showed preference for Na^+^ conductance over that of other cations whereas the class II HKT transporters select either Na^+^ and/or K^+^ (Mäser et al., 2002b; Almeida et al., 2014). A variety of plant species have been shown to have HKT-type transporters (Schachtman and Schroeder, 1994; Nieves-Cordones et al., 2016), and their functions have been studied in different ways. A heterologous expression system of *TaHKT2;1* in wheat, for example, showed dual modes of action. In other words, when the Na^+^-K^+^ exchange rate was low, it acted as a Na^+^- K^+^ symporter, while when the exchange rate was high, it acted as a Na^+^-uniporter(Rubio et al., 1995; Gassmann et al., 1996). Transgenic wheat plants increased in growth under salinity when *TaHKT2;1* was knocked down genetically (Laurie et al., 2002), indicating that TaHKT2;1 can be a Na^+^-uptake pathway in wheat roots. Several heterologous expression systems have also characterized Arabidopsis *AtHKT1;1* and Rice *OsHKT2;1* as Na^+^-uniporters (Uozumi et al., 2000; Horie et al., 2001; Garciadeblás et al., 2003). The genetic mutations of *AtHKT1;1* in Arabidopsis roots showed that it does not mediate Na^+^ influx (Mäser et al., 2002a; Berthomieu et al., 2003; Horie et al., 2006); however, OsHKT2;1 mediates Na^+^ uptake in roots with low K^+^ levels (Horie et al., 2007). The salt-tolerance mechanisms of monocotyledonous halophytes can be used as a tool to improve salt tolerance crops.

It has been increasingly clear how halophytes have adapted to high salinity conditions over the past few decades. The morphological and biochemical adaptations of halophytes allow them to cope with high soil salinity (Flowers et al., 2010). *Salicornia europaea* is a salt marsh halophyte belonging to the *Amaranthaceae* family that is one of the most salt-tolerant plants on the planet(Patel, 2016). *S. europaea* imparted a higher salt tolerance. In *S. europaea*, salt tolerance may be attributed to its ability to restrict Na^+^ influx unidirectionally to roots, leading to a large concentration gradient between roots and shoots (Bressan et al., 2013). As the uptake of K^+^ is maintained in *S. europaea* roots, it has been proposed that the endodermal barrier to Na^+^ restricts Na^+^ uptake. The molecular identity of the channel or transporter associated with the restriction of unidirectional Na^+^ influx has not been disclosed (Xu et al., 2016).

In this study, we cloned a novel gene encoding a potassium transporter of the HKT type from *S. europaea* (SeHKT1;2). The gene expression pattern of *SeHKT1;2* under K^+^-starvation and salt stress was analyzed and its function was characterized in yeast and Arabidopsis.

## Materials and methods

### Plant materials, growth conditions, and stress treatments

Seeds of *S. europaea* were collected from an alkaline soil area of Xinjiang, China. Plants were germinated in tap water for 14 days and then transferred to half-strength Hoagland’s nutrient solution and allowed to grow for another 14 days before stress treatments. The temperatures of the growth chamber were maintained at 28°C during the day (16 h) and 22°C at night (8 h) under a photoperiod (350-400 µmol m^-2^ s^-1^). For the salt-stress treatment, NaCl was added to half-strength Hoagland’s nutrient solution with 0 mM, 10 mM, 200 mM, 500 mM, and 800 mM separately. For the K^+^-starvation treatment, KCl was removed from the standard solution. Under 200 mM NaCl treatment, plants tissue samples were harvested at 0, 6, 24, 48, 72, and 120 h after stress treatments and preserved at –80°C for further analyses.

### Cloning of SeHKT1;2 from *S. europaea*

Total RNA was extracted from the shoots of *S. europaea* using RNeasy plant extraction Mini Kit (Qiagen, Germany) according to the manufacturer’s instructions. Total RNA was reverse transcribed by using an oligo dT primer and MMLV-reverse transcriptase (TaKaRa, China). Full-length cDNA was obtained using 5’- and 3’-RACE (Rapid Amplification of cDNA Ends) techniques. The partial cDNA fragment was amplified by PCR, using a degenerate primer (**Table S1**) deduced from the conserved regions of HKT and a reverse transcription product as a template. The full-length cDNA of *SeHKT1;2* was amplified using primers (**Table S1**). The PCR product was sub-cloned into the pMD-19 T vector and sequenced.

### Bioinformatic analysis of *SeHKT1;2* sequence

Transmembrane domains were predicted by DeepTMHMM (https://biolib.com/damgaard/DeepTMHMM-testing). A maximum-likelihood phylogenetic tree of plant HKT proteins was constructed using the MEGA program (version 7.0, Auckland, NewZealand). The accession numbers of genes utilized in the present study are listed in **Table S2**. The protein secondary structure was predicted using NetSurfP - 3.0 online program(Høie et al., 2022). The 3D model of SeHKT1;2 protein was build using SWISS-MODEL online program (Waterhouse et al., 2018).

### RNA extraction and cDNA synthesis for real-time RT-PCR

For real-time RT-PCR, total RNA was extracted from the shoots and roots of *S. europaea* subjected to 0 mM Na^+^, 10 mM Na^+^, 200 mM Na^+^, 500 mM Na^+^, and 800 mM Na^+^, K^+^-starvation, time-course using the RNeasy plant extraction Mini Kit (Qiagen, Germany) according to the manufacturer’s instructions. Two micrograms of DNase-treated RNA were transcribed to cDNA using the Reversal Transcription Reagent Kit (TaKaRa) following the manufacturer’s instructions. The cDNA was diluted 10 times and 1µl of the diluted cDNA was used as the template in each well for quantitative real-time PCR analysis. The cDNA was amplified using Power SYBR Green PCR Master Mix (Applied Biosystems, USA) on CFX96 Real-Time PCR Detection System (Bio-Rad, USA). A *α-tubulin* gene from *S. europaea* has been used as internal reference (Lv et al., 2012; Ma et al., 2013; Xiao et al., 2015) and served as an internal standard to normalize the expression data for the *SeHKT1;2* gene. The qRT-PCR was performed using sequence specific primers (**Table S2**). The thermal profile for qRT-PCR was as follows: 2 min at 95°C, 40 cycles of 15 s at 95°C and 30 s at 60°C, and a melting curve protocol (plates read when increased 0.5°C every 5 s from 65°C to 95°C). The melting curve verified the amplification specific and confirmed that there were no primer dimers. All of the samples were run with replicates. The threshold cycle (Ct) values were measured according to the setting of an auto-calculated baseline threshold in Bio-Rad CFX Manager software (Bio-Rad, USA).

### Functional characterization of *SeHKT1;2* in yeast

The ORF of *SeHKT1;2* was inserted into the yeast protein expression vector pYES2. To test the Na^+^ Absorption and efflux function of *SeHKT1;2*, the *Saccharomyces cerevisiae* yeast mutant strain AXT3K (*enal::Hls3::ena4,nhal::LEuZ,nhxl::KanMx4*), which lacks the main plasma membrance Na^+^ transporters, and G19 (*MATa ade2ura3leu2his3trp1 ena1Δ::HIS3Δ::ena4Δ*) disrupted in the *ENA1-4* genes encoding Na*^+^* export pumps were used. The plasmids were introduced by PEG/LiAc method. Positive transformants were selected on Ura-selective medium [0.67% (w/v) yeast nitrogen base without amino acids, 0.077% (w/v) DO supplement-Ura, 2% (w/v) galactose, and 1.5% (w/v) agar]. Growth at variable Na^+^ concentrations with 0, 25, 30, 60, 100 or 150 mM for AXT3K and 0, 60, 100, 150, 300 mM for G19 were tested in arginine phosphate (AP) medium [8 mM phosphoric acid, 10 mM L-Arginine, 2 mM MgSO_4_, 0.2 mM CaCl_2_, 2% glucose, plus vitamins and trace elements, and 1.5% (w/v) agar, pH 6.5].

Meanwhile, Yeast (Saccharomyces cerevisiae) strain CY162 (*MATa, △trk1, trk2:: pCK64, his3, leu2, ura3, trp1, ade2*), which is a K^+^-uptake-defective mutant and cannot grow without supplement K^+^ was used to test K^+^-uptake function of *SeHKT1;2* gene. Positive transformants were selected on Ura-selective medium with 100 mM KCl. Yeast growth experiments were performed on arginine-phosphate (AP) medium with added K^+^ (1 mM, 10 mM) and supplemented with 100 mM, 150 mM and 300 mM Na^+^ concentrations. Control experiments were performed with the yeast modified with vector pYES2 and pYES2*-SeHKT1;2* growing under 100 mM KCl.

For the yeast growth test experiment, all transformed yeasts were cultured overnight at 30°C in AP medium until the OD_600_ reached 0.8, and 10-fold serial diluted cultures were incubated on AP plates containing the indicated concentrations of K^+^ and Na^+^. The plates were incubated at 30°C for 5 days. Control experiments were performed with the yeast wild type modified with vector pYES2.

### Generation of transgenic Arabidopsis plants over-expressing the *SeHKT1;2* gene

The coding regions of *SeHKT1;2* was sub-cloned into the plant transformation binary vector pBI121 (Clontech, Japan) under the control of CaMV35S promoters. The constructs were introduced into *Agrobacterium tumefaciens* strain GV3101 and Arabidopsis salt-hypersensitive mutant *sos1* was transformed by the floral dip method (Clough and Bent, 1998). Transgenic plants were selected on half-strength Murashige & Skoog medium containing 25 mg/L kanamycin. qRT-PCR showed the introduced gene transcript in 10 out of 15 lines of *SeHKT1;2* with varied expression levels among the lines. Two lines transgenic plants with higher expression level were used for phenotype assays of salt stresses. All of the lines used in these experiments were homozygous. Wild type and transgenic *Arabidopsis* seeds were surface-sterilized with 70% (v/v) ethanol for 2 min and 1% (v/v) NaCl solution for 15 min. The seeds were rinsed three times with water and sown on an MS medium containing 1.5% (w/v) sucrose, 0.8% (w/v) agar, and 50 mg/L kanamycin. Seven-day-old seedlings were transferred from germination medium to soil, supplemented with the indicated amounts of 300 mM NaCl after ten days.

## Results

### Identification of *SeHKT1-like* gene from *S. europaea*

**Figure 1:**
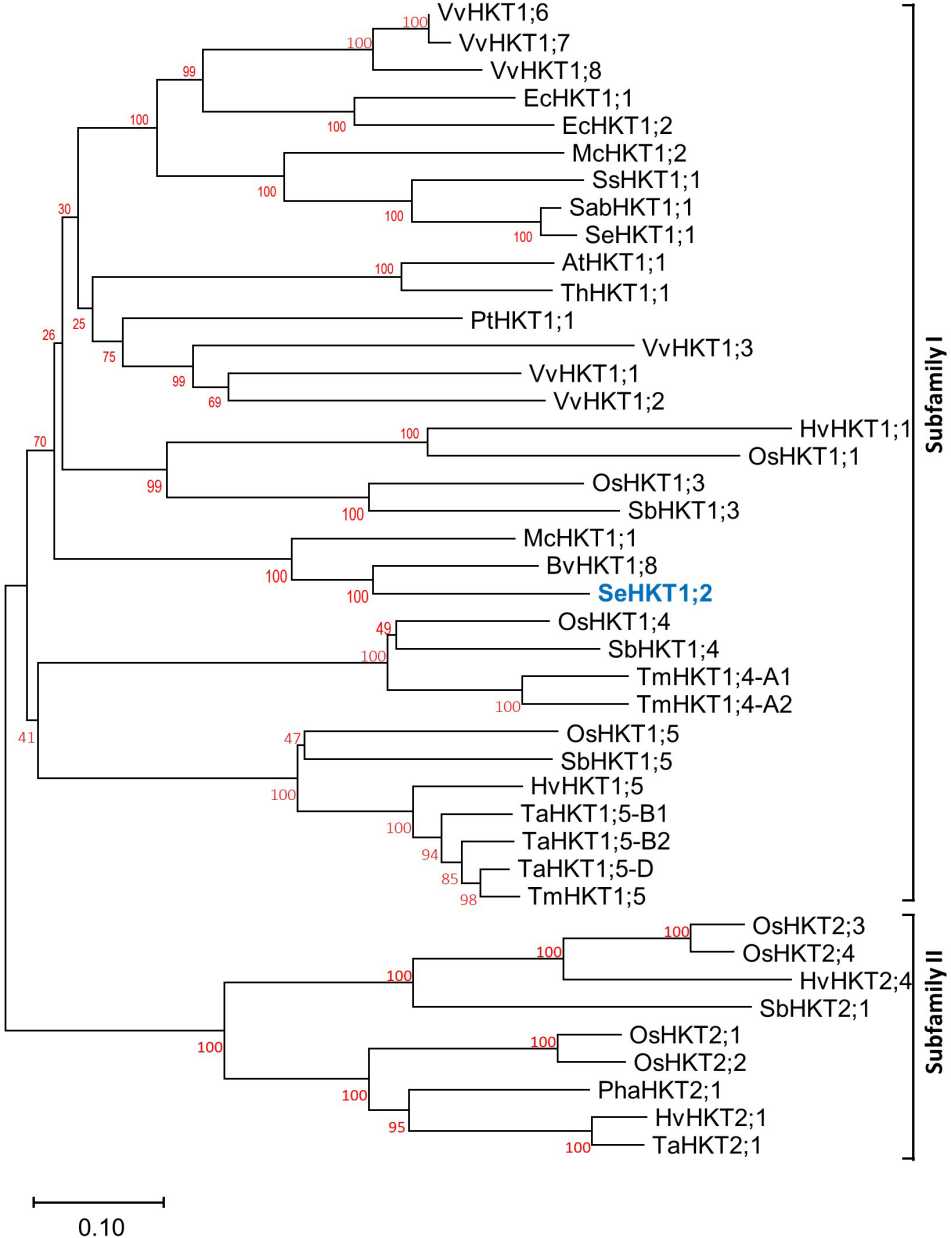
The phylogenetic relationship of SeHKT1;2 with HKTs from other plant species. Full length of HKT proteins form different plant species were used and the tree was generated by using neighbor-joining method with 1000 bootstrap replicates. Accession numbers and species for all sequences are listed in **Supplemental Table S1**.

By using degenerate primers deduced from several *HKT* sequences and standard reverse transcription (RT)-PCR methods, a cDNA homolog of an HKT high-affinity K^+^ transporter from *S. europaea* was cloned. The length of the amplified cDNA fragment was about 1500-bp long. The full-length cDNA was obtained by 5′ and 3′ RACE (Rapid Amplification of cDNA Ends) which contains an ORF of 1611-bp long (**Figure S1A**). The translated amino acid sequence of this gene exhibited 44.31% amino acid sequence identity with SeHKT1;1 (AKS12114.1) (**Figure S1B**) therefore it was designated as *SeHKT1*;2 (AKS2645929). Subsequently the evolutionary relationships of SeHKT1;2 with HKT proteins from other plant species. An unrooted phylogenetic tree was generated based on full-length amino acid sequences. Results showed that SeHKT1;2 was grouped into subfamily I of HKT transporters (**Figure 1**).

### Structural characteristic of SeHKT1;2

**Figure 2:**
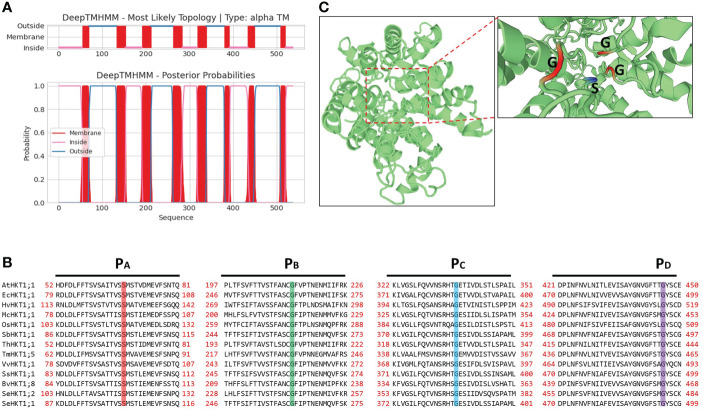
Structure analysis of SeHKT1;2 protein. **(A)** Prediction of the transmembrane domain of SeHKT1;2 protein. Blue lines are pore loop regions, and red boxes are transmembrane regions. **(B)** Multiple sequence alignment of the four conserved selectivity-filter-pore regions of HKT proteins. Amino acid sequences were aligned using Clustal W and visualized using BioEdit. Conserved residues in pore loops were indicated with colored lines. **(C)** Predicted 3D model of SeHKT1;2 protein. The first P-loop (PA, S/G) and the other P-loop (PB-D, G) are shown in magenta and orange (Color figure online).

The *SeHKT1;2* encoding a 536 amino acid polypeptide with 8 predicted transmembrane domains (**Figure 2**). SMART domain architecture analysis showed that SeHKT1;2 protein has single TrkH conservative domain (pfam ID: PF02386) between amino acids 70-524 (**Figure S2A**). The secondary structure of the SeHKT1;2 protein was predicted, and it was found to have a complex helical folding structure (**Figure S2B**). The 3D models of SeHKT1;2 showed the presence of three glycine residues (Gly244, Gly368 and Gly480) and one serine residue (Ser119) forming an ion selectivity filter, which belong to subfamily I (**Figures 2**, **S3**).

### Tissue specific expression of *SeHKT1;2* gene

**Figure 3:**
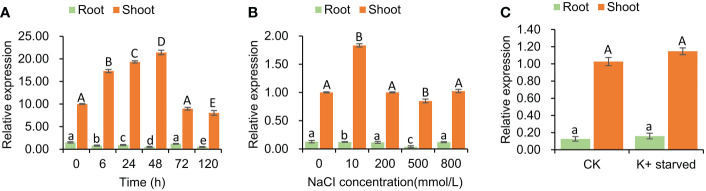
Tissue specific expression of *SeHKT1;2* gene. **(A)** Time course expression level of *SeHKT1;2* gene in shoots and roots. **(B)** The relative expression level of *SeHKT1;2* gene under 0, 10, 200, 500, 800 mmol NaCl concentration treatment. Plant samples were taken after 72h of NaCl treatment. **(C)** Expression pattern of *SeHKT1;2* gene under K^+^ starved condition. After the seedlings were pre-cultured with standard Hoagland nutrient solution for 30 days, the seedlings were divided two groups. One group were continue cultured with standard Hoagland nutrient solution as CK group. Another group were cultured with $\text{N}{\text{O}}_{3}^{\;-}$ - free Hoagland nutrient as K^+^-starved group. Plant samples were taken after 72h of K^+^ starvation treatment. *α-tubulin* was used as the internal reference for data processing. Values indicated the mean and standard deviation of biological repetitions (n = 3).

The expression pattern of *SeHKT1;2* gene under the salt stress conditions was determined. The *SeHKT1;2* gene expression patterns in *S. europaea* seedlings were completely different between shoots and roots. In a time-course experiment, the *SeHKT1;2* was induced by NaCI (200mM) and reached the peak at 48h in shoot, but it was gradually reduced in root (**Figure 3**). Subsequently, we quantified expression level of *SeHKT1;2* gene under the presence of NaCI. As a result, the 10 mM NaCI significantly increased the expression of *SeHKT1;2* in shoot, but it remained same in the presence of high concentration (200mM, 500mM, 800 mM) of NaCI (**Figure 3**). In addition, we also examined the *SeHKT1;2* gene expression under K^+^ starved condition. In our experiments, *SeHKT1;2* showed a very low expression level in roots while showed 10 folds higher in shoots (**Figure 3**). The expression of *SeHKT1;2* in root was not affected by NaCI.

### Functional characterization of SeHKT1;2 in yeast

**Figure 4:**
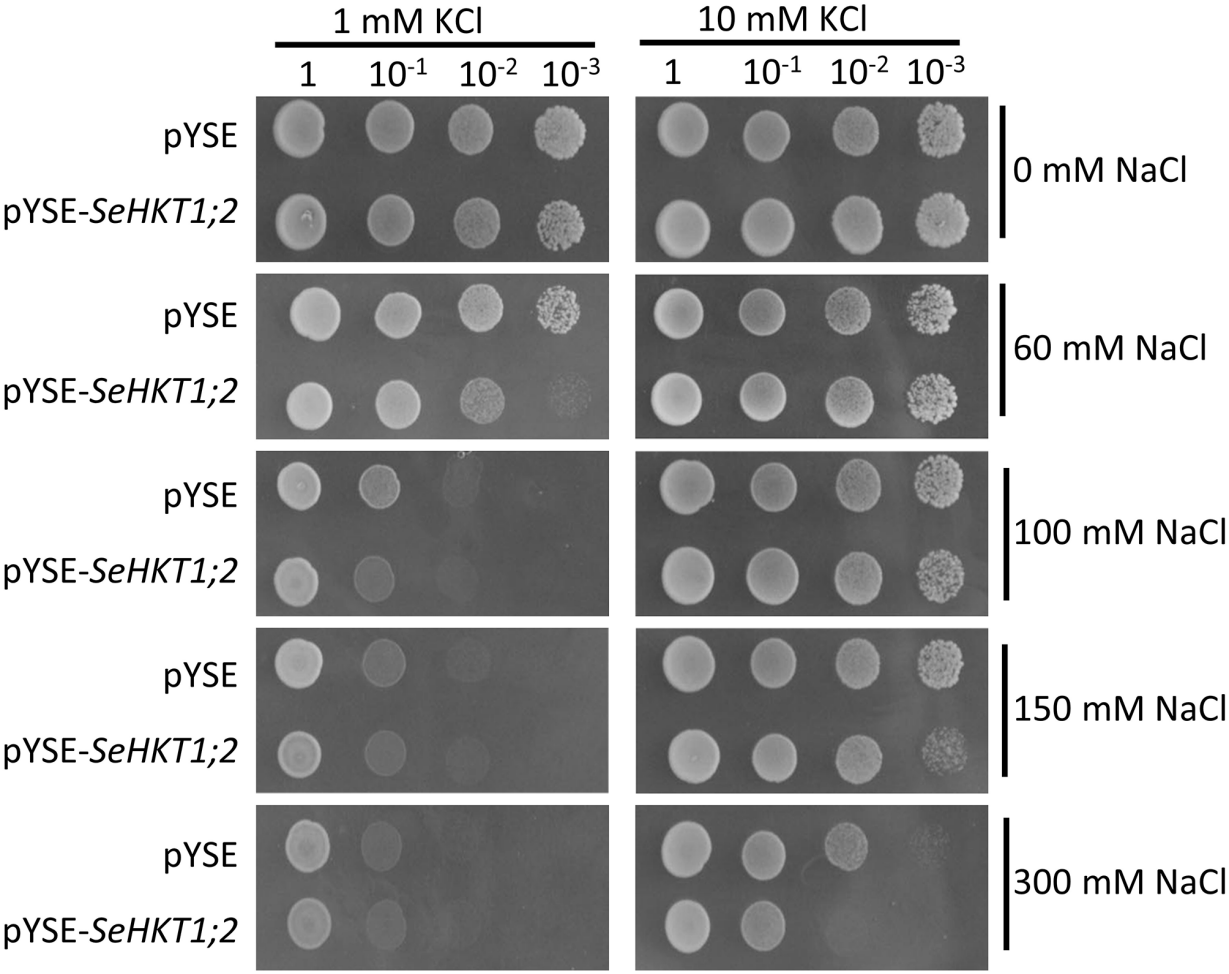
Functional characterizations of *SeHKT1;2* in G19 yeast strain. Growth of G19 yeast cells transformed by empty vector (pYSE) control and pYSE-SeHKT1;2. Each G19 transformants on solid arginine phosphate (AP) medium supplemented with various concentrations (1mM,10 mM) of KCl with combination of different salt concentrations (0, 60, 100, 150, and 300 mM NaCl) were incubated at 30°C for 3 days. Numbers (1, 10^-1^, 10^-2^, 10^-3^) on the top of each panel indicated serial dilutions of yeast cells placed on the medium.

Previous functional characterization in yeasts showed that SaHKT1 from the *Suaeda salsa* functions as a Na^+^ selective transporter (Wang et al., 2020). To determine whether SeHKT1;2 is involved in Na^+^ uptake, we expressed *SeHKT1;2* proteins in a Na^+^-sensitive mutant yeast strain, G19 (Quintero et al., 1996). Results showed that under the low concentration of K^+^ (1Mm), the increasing of Na^+^ concentration in the growth medium caused severe growth defects in both SeHKT1;2-expressing cells and empty vector-harboring control cells (**Figure 4** left panel). Interestingly, higher K^+^ concentration (10mM) improved the growth of both control and SeHKT1;2-expressing cells (**Figure 4** right panel). Moreover, the higher concentration (300mM) of Na^+^ restricted cell growth of both control and SeHKT1;2-expressing cells (**Figure S4**). These data indicate that Na^+^ toxicity was reduced by K^+^ uptake, and that cells expressing SeHKT1;2 has a selective advantage under K^+^ limitation suggesting that SeHKT1;2 have a higher affinity for K^+^.

**Figure 5:**
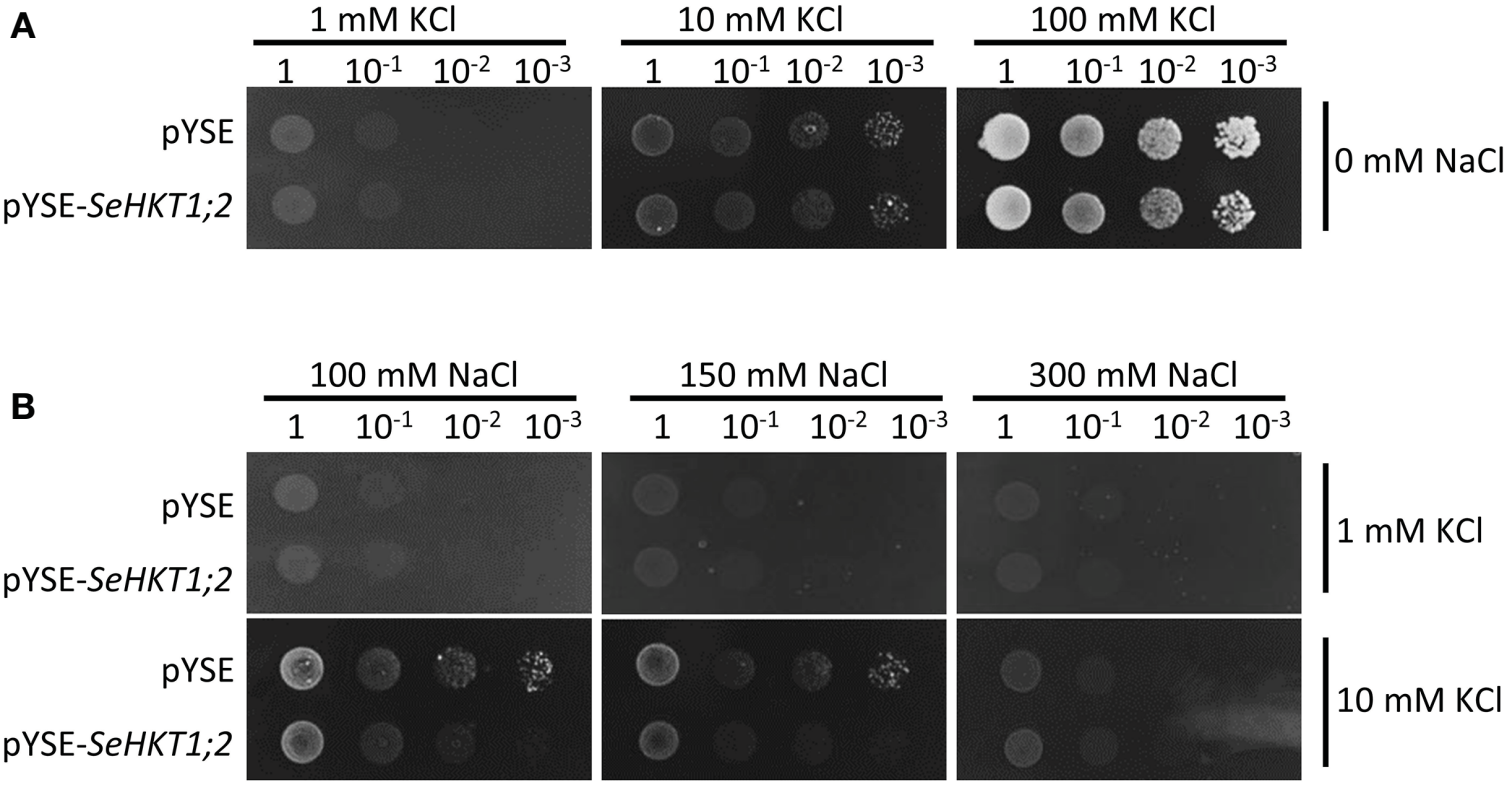
Complementation assay in yeast CY162 strain. **(A)** Growth of yeast strain CY162 cells harboring the empty vector (pYSE) control and pYSE-SeHKT1;2. Growth of each CY162 transformant on solid arginine phosphate (AP) medium supplemented with various concentrations (1mM,10 mM, 100 mM) of KCl and 0 mM NaCl incubated at 30°C for 3 days. Numbers (1, 10^-1^, 10^-2^, 10^-3^) on the top of each panel indicated serial dilutions of yeast cells placed on the medium. **(B)** Growth status of the yest cell lines in **(A)** on AP medium containing different concentration of KCl (1 mM,10 mM) and NaCl (100mM,150mM,300mM). Each yest cell lines were incubated at 30°C for 3 days. Numbers (1, 10^-1^, 10^-2^, 10^-3^) on the top of each panel indicated serial dilutions of yeast cells placed on the medium.

To investigate the transporter activity of SeHKT1;2, we performed complementation assay by ectopic expression of *SeHKT1;2* in the K^+^ transporter-deficient yeast strain CY162 (Anderson et al., 1992). We observed no significant differences between *SeHKT1;2* and empty vector control in their growth supplied with various concentration of KCl, and both grew well on the medium containing 100 mM KCI (**Figure 5A**). This indicates that *SeHKT1;2* failed to complement K^+^ uptake. Additionally, the transformants growth was completely impacted as the non-transformed strain when K^+^ and Na^+^ were applied in combined form, in the CY162 with increased K^+^ (10Mm KCI) in combination with Na^+^, the transformants growth was somehow recovered (**Figure 5B**). These results suggested that a trend toward K^+^ selectivity by SeHKT1;2.

### Heterologous expression of *SeHKT1;2* in Arabidopsis *sos1* mutant

**Figure 6:**
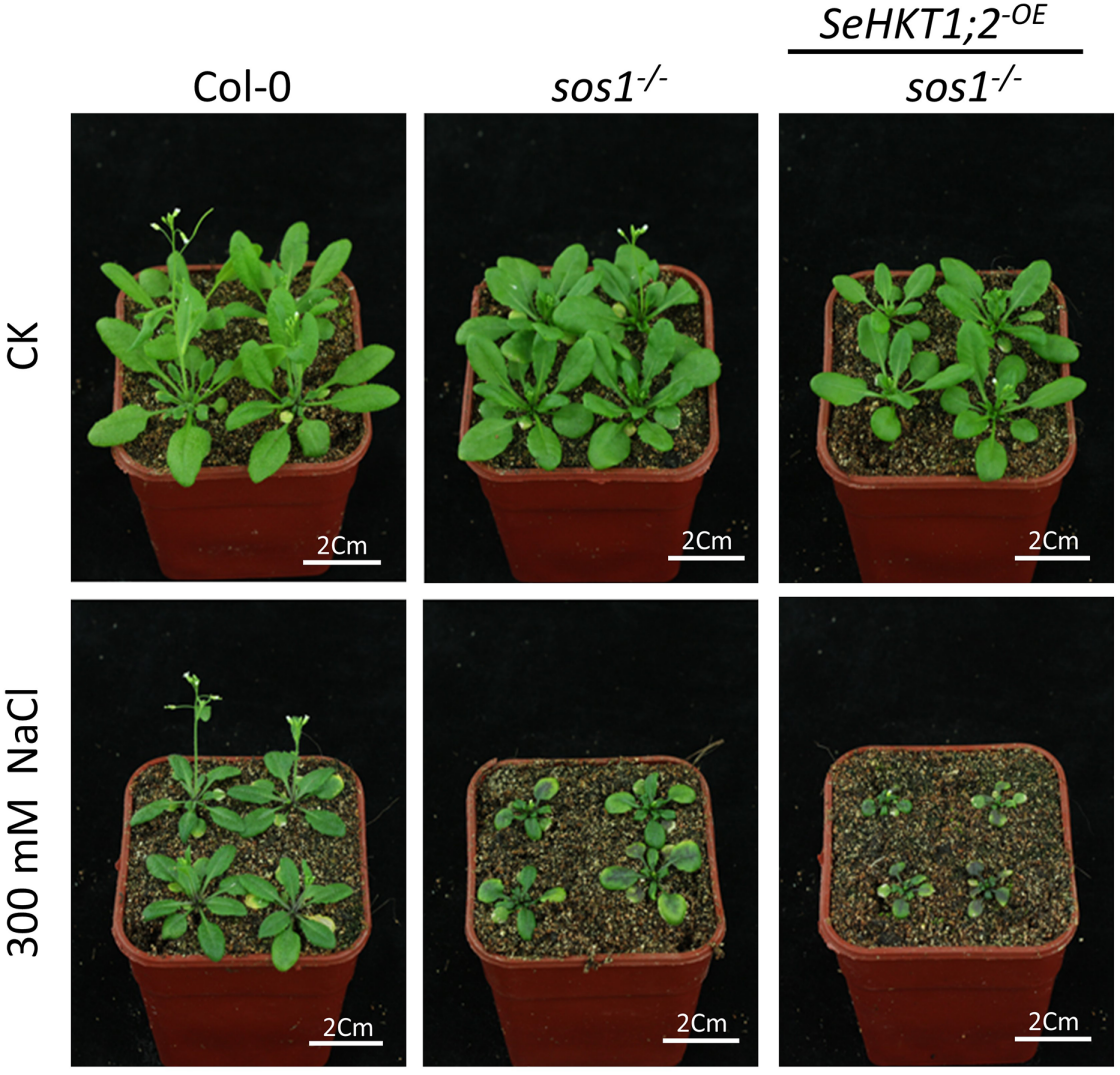
Phenotypes of transgenic and wild type Arabidopsis lines under salt stress. The *sos1* mutant Arabidopsis were infiltrated by *Agrobacterium tumefaciens* harbored the *SeHKT1;2* construct. Four-week-old Arabidopsis plants were treated with watered with 300 mM NaCl for 14 days.

In an attempt to validate the function of *SeHKT1;2* in salt tolerance, we over expressed the *SeHKT1;2* in Arabidopsis *sos1* mutants. As expected, the whole phenotypes of *SeHKT1;2*-expressing in *sos1* and wild type Col-0 plants showed the same growth vigor and had no obvious difference under the normal condition. The growth of *sos1* plant, however, was restricted by imposing of salt stresses (300 mM NaCl) and attenuated by overexpression of *SeHKT1;2* (**Figure 6**). Interestingly, the overexpression of *SeHKT1;2* cannot restore the salt tolerance of *sos1* mutant plant.

## Discussion

HKT transporters are one of the well-studied cation transporters in plant species. From an *S. europaea* halophyte, a homolog of HKT, *SeHKT1;2*, was isolated and the amino acids at the four pore-loop conservative sites of SeHKT1;2 protein are Ser-Gly-Gly-Gly, which belong to subfamily I (**Figure 2**). The presence of a Ser (serine) in the first pore-lope regions of HKT protein is associated with Na^+^ selectivity, and is associated with Na^+^ and/or K^+^ selectivity only when the serine at this position is changed to Gly (glycine) (Horie et al., 2009). Although the rice OsHKT2;1 has Ser residue at the filter position of the first P-loop but exhibits features of class I transporters such as poor K^+^ permeability (Horie et al., 2001; Yao et al., 2010).

The AtHKT1, EsHKT1;2(TsHKT1;2) and EpHKT1;2 belong to subfamily I and contain the conserved serine residue in the first pore-lope region (Ali et al., 2012; Ali et al., 2018). Unlike the AtHKT1, the EsHKT1;2 and EpHKT1;2 show significantly higher affinity for K^+^ than for Na^+^ (Ali et al., 2018). Presence of aspartic acid residue (D) in the second pore-loop domain of EpHKT1;2 (D207,D238) and EsHKT1;2 (D205,D236) determines their cation specificity (Na^+^ or K^+^) (Ali et al., 2016; Ali et al., 2018) suggesting that glycine residues are not the only amino acids involved in K^+^ transport in HKT proteins. SeHKT1;2 contains asparagine residue (N) instead of aspartic acid residue (D) in the second pore-loop domain at the corresponding positions (D242, D273) in which same with AtHKT1 (**Figure S5**) and supposed to has functional similarity with AtHKT1. In contrast, SeHKT1;2 exhibited higher affinity for K^+^ (**Figures 4**, **5**), whereas AtHKT1 showed a higher affinity for Na^+^. Although SeHKT1;2 shared similarity with AtHK1 in key amino acids but functionally different form AtHK1 indicating that other structural differences, presently unknown, may enhance K^+^ uptake of SeHKT1;2.

Plant HKT gene expression is always affected by salt stresses. *PtHKT1;5* from a halophytic grass, *Puccinellia tenuiflora*, was up regulated in root cells by salt (NaCI) stress. Unlike *PtHKT1;5*, the *SsHKT1;1* from another halophyte, *Suaeda salsa*, was downregulated under NaCl treatment. It seems that the expression of HKT1 is up-regulated in salt-excluding while down-regulated salt-accumulating halophyte species under salt stress. *S. europaea* is a salt-accumulating halophyte, and transcript of *SeHKT1;2* in root was also decreased by the time under salt stress (**Figure 3**). In contrast, salt stress induced the *SeHKT1;2* gene expression in shoot of *S. europaea* (**Figure 3**). The differential expression of *SeHKT1;2* gene in root and shoot may reduce Na^+^ retrieval from the xylem and facilitate Na^+^ transport into its shoots so *S. europaea* could adjust to high external salinity and exhibit better growth under saline environment. Hence, SeHKT1;2 is key protein in salt accumulation in *S. europaea*.

**Figure 7:**
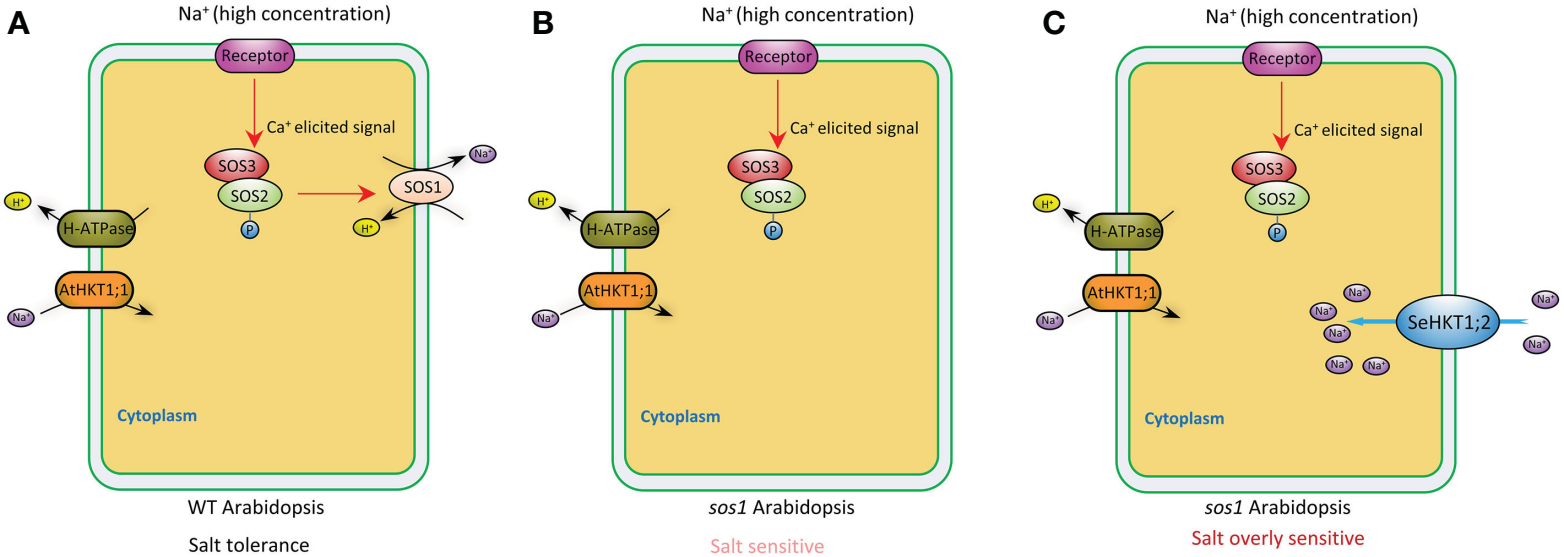
A proposed model depicting the putative function of SeHKT1;2 contributing to Na^+^ uptake across the plasma membrane. In wild type Arabidopsis, high Na^+^ concentration elicits a Ca2^+^ signal that activates SOS3-SOS2 complex, which in turn stimulates cytoplasmic Na^+^ efflux activity of SOS1 across the plasma membrane **(A)**. Efflux of cytoplasmic Na^+^ was reduced in *sos1* Arabidopsis, making the plants salt sensitive **(B)**. Na^+^ uptake across the plasma membrane by SeHKT1;2 increased the cytoplasmic Na^+^ concentration, making the plants salt overly sensitive **(C)**.

Plants maintain sodium homeostasis by Salt Overly Sensitive (SOS) pathway (Yang and Guo, 2018). AtSOS1 is the key sodium transporter in Arabidopsis SOS pathway. The *sos1* mutant Arabidopsis plants are even more sensitive to Na^+^ stresses compared with *sos3* mutant plants (Zhu et al., 1998; Shi et al., 2000). A previous study demonstrated that Na^+^ efflux rate significantly decreased by 16% in *sos1* compared with WT (Wang et al., 2019). In our study, *sos1* exhibited more sensitivity to salt stress and heterologous expression of *SeHKT1;2* could not rescue the *sos1* mutant plants but increased the sensitivity of the *sos1* mutant plants (**Figure 6**). In the *sos1* mutant plants the impairment of AtSOS1 mediated efflux of cytoplasmic Na^+^ resulting in accumulation of cytoplasmic Na^+^ and showed salt sensitivity (**Figures 6**, **7**). The salt overly sensitive phenotype caused by heterologous expression of SeHKT1;2 can be explained that the *SeHKT1;2* in *sos1* Arabidopsis influx Na^+^ so that further accumulate cytoplasmic Na^+^ and eventually inhibits plant growth and increases salt sensitivity of *sos1* plant (**Figures 6**, **7**). This study suggested that SeHKT1;2 play very important roles in synergistically regulating Na^+^ homeostasis by controlling Na^+^ transport in Arabidopsis.

## Data availability statement

The original contributions presented in the study are included in the article/**Supplementary Material**. Further inquiries can be directed to the corresponding authors.

## Author contributions

The experimental design, data analyzation, manuscript organization were completed by JM and YH and ZP were assistant with RNA quantification. JM and LW conceived the project, supervised the analysis, and critically revised the manuscript. XF revised the manuscript. All authors contributed to the article and approved the submitted version.
