# Supplementary figures and images for: A novel high-affinity potassium transporter SeHKT1;2 from halophyte Salicornia europaea shows strong selectivity for Na+ rather than K+
Source: Front Plant Sci. 2023 Feb 20;14:1104070. doi: 10.3389/fpls.2023.1104070 (PMC9986455; doi:10.3389/fpls.2023.1104070)

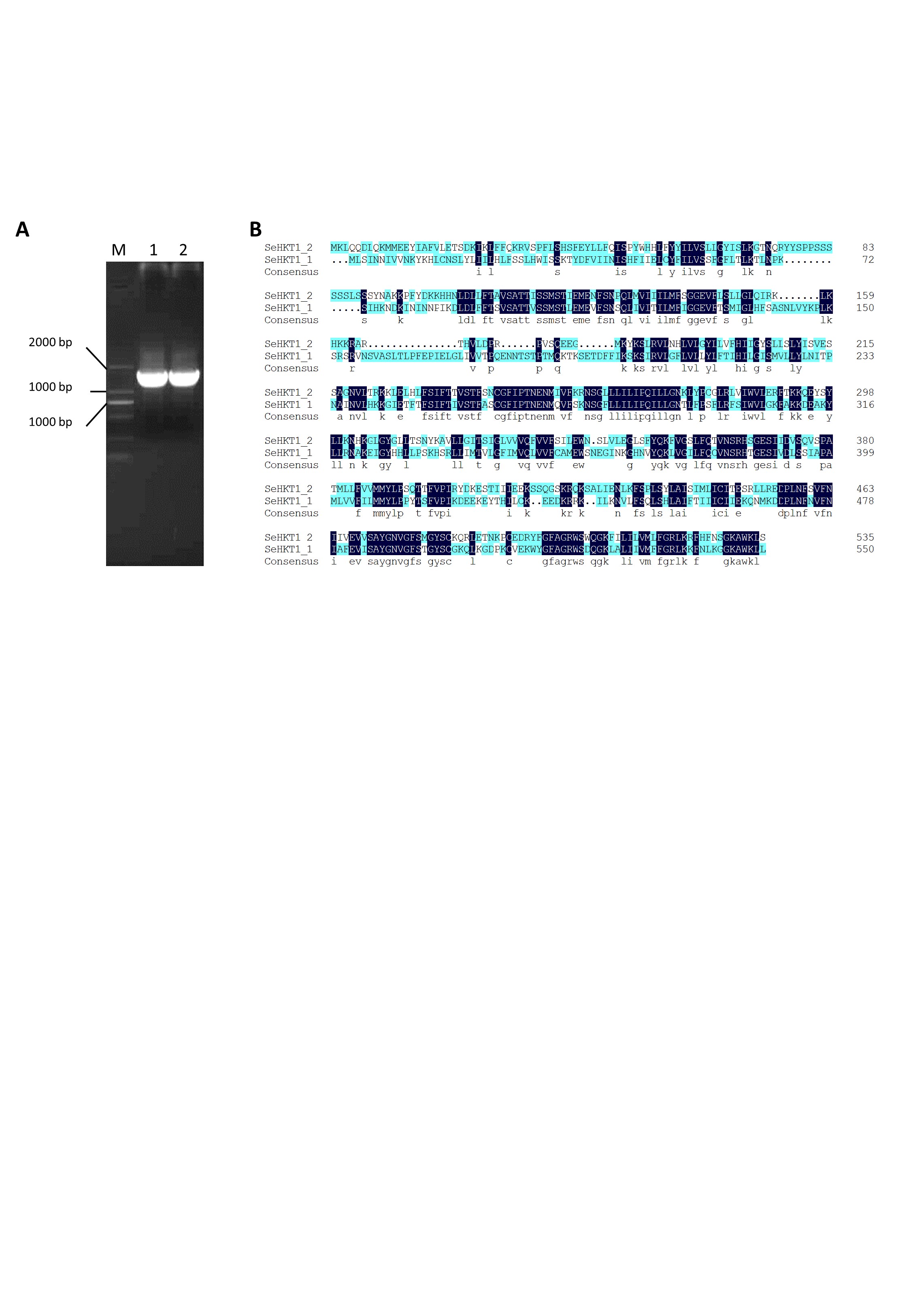

Supplement: Supplementary file 1 [file Image_1.jpeg]

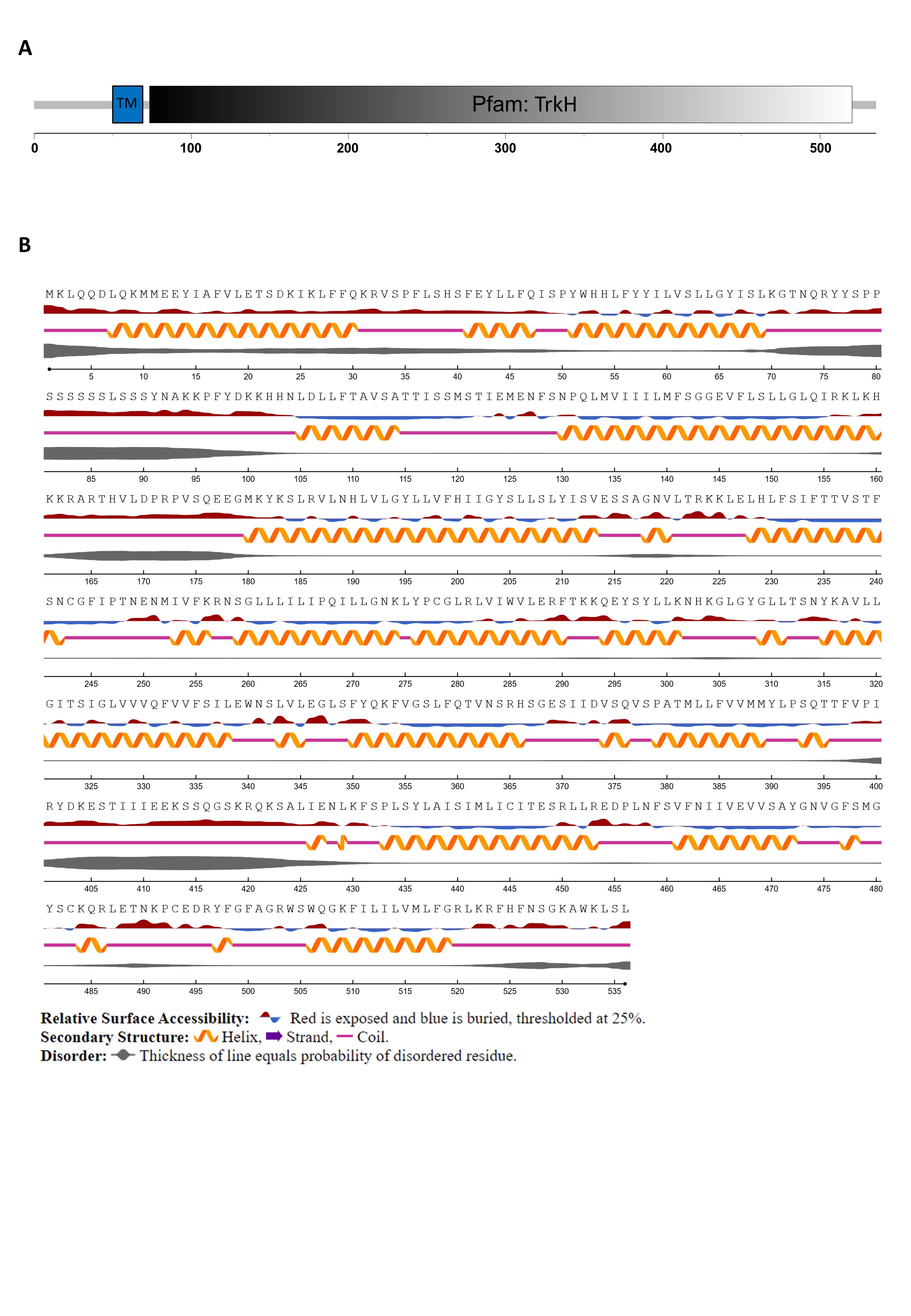

Supplement: Supplementary file 2 [file Image_2.jpeg]

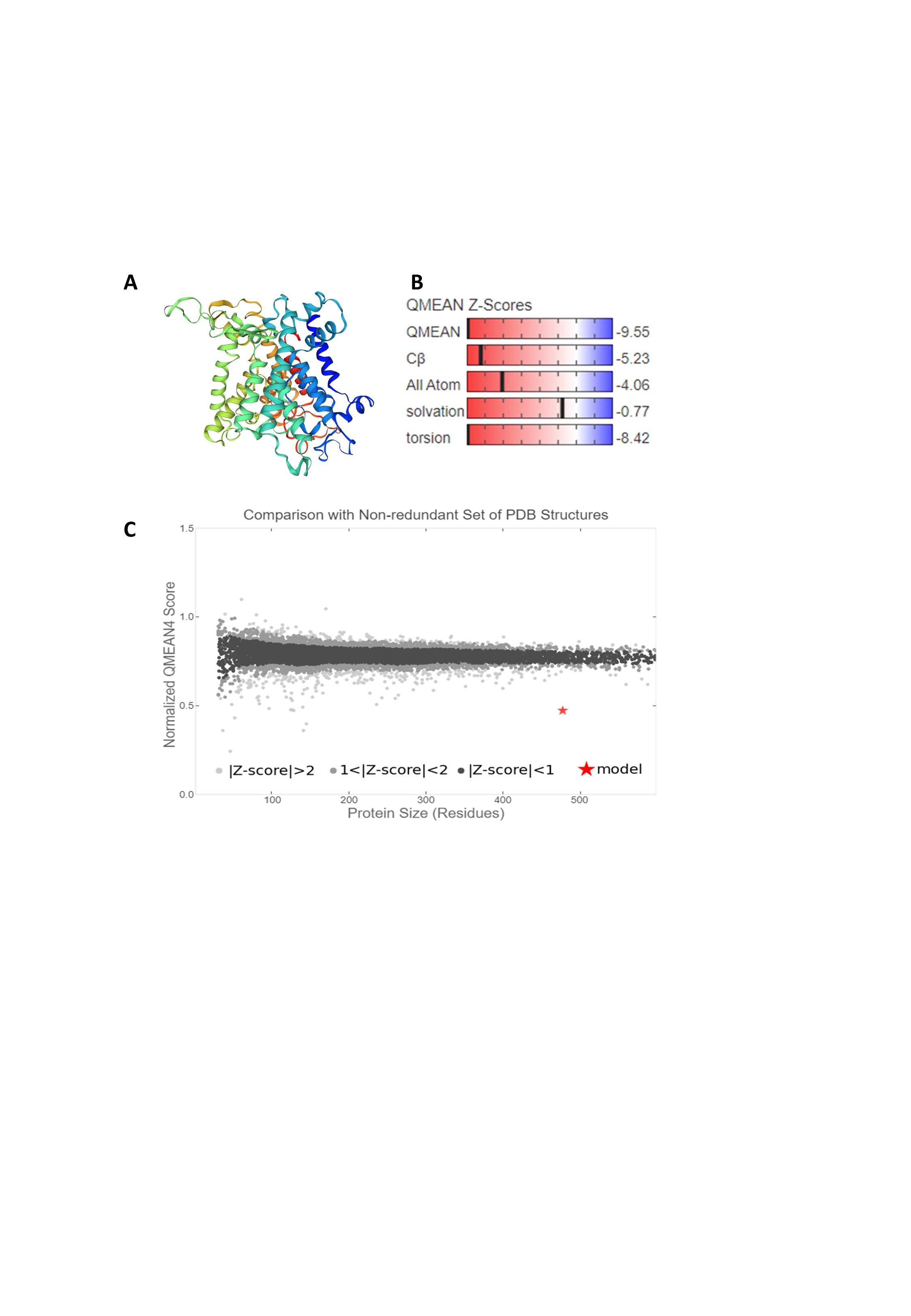

Supplement: Supplementary file 3 [file Image_3.jpeg]

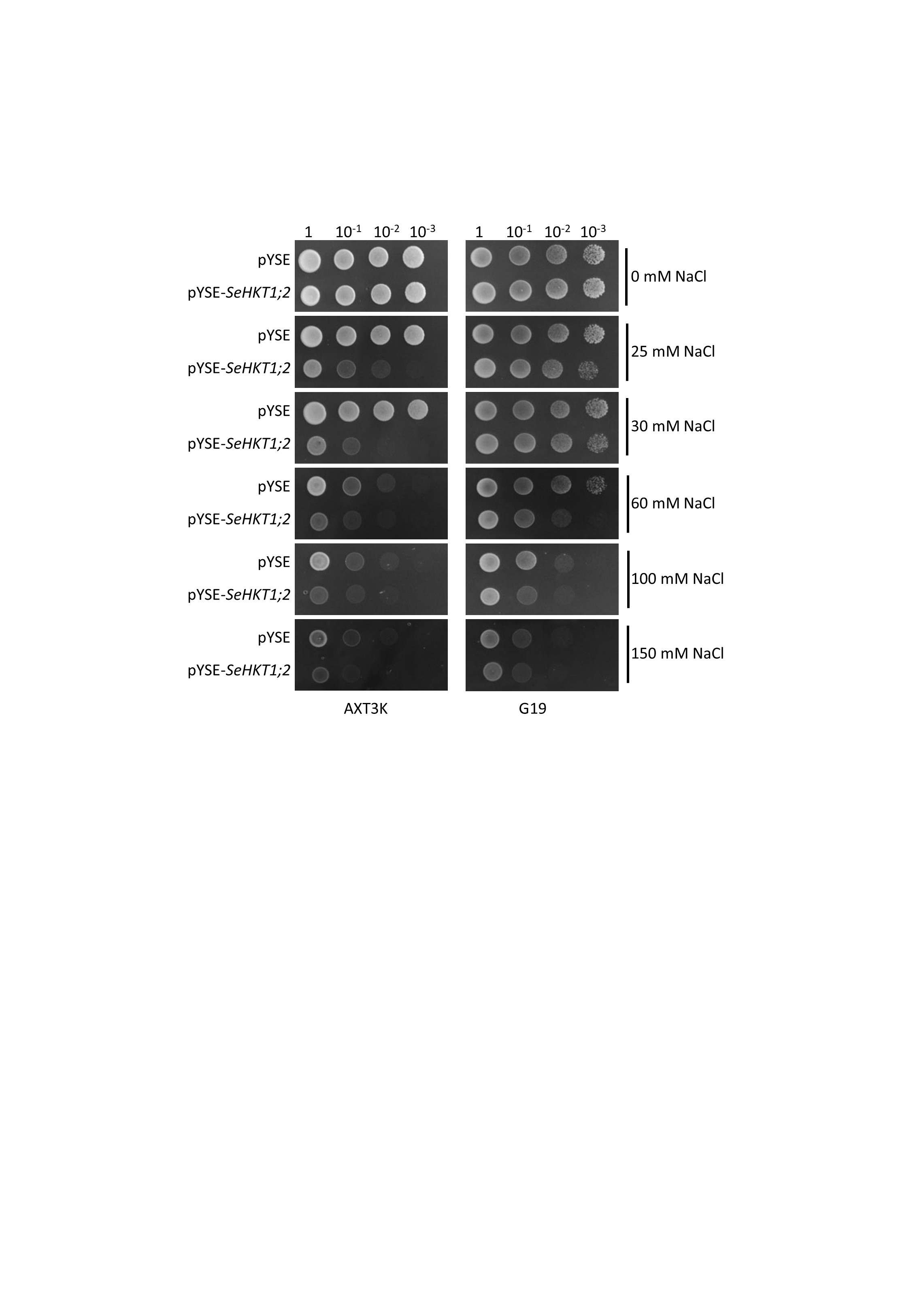

Supplement: Supplementary file 4 [file Image_4.jpeg]

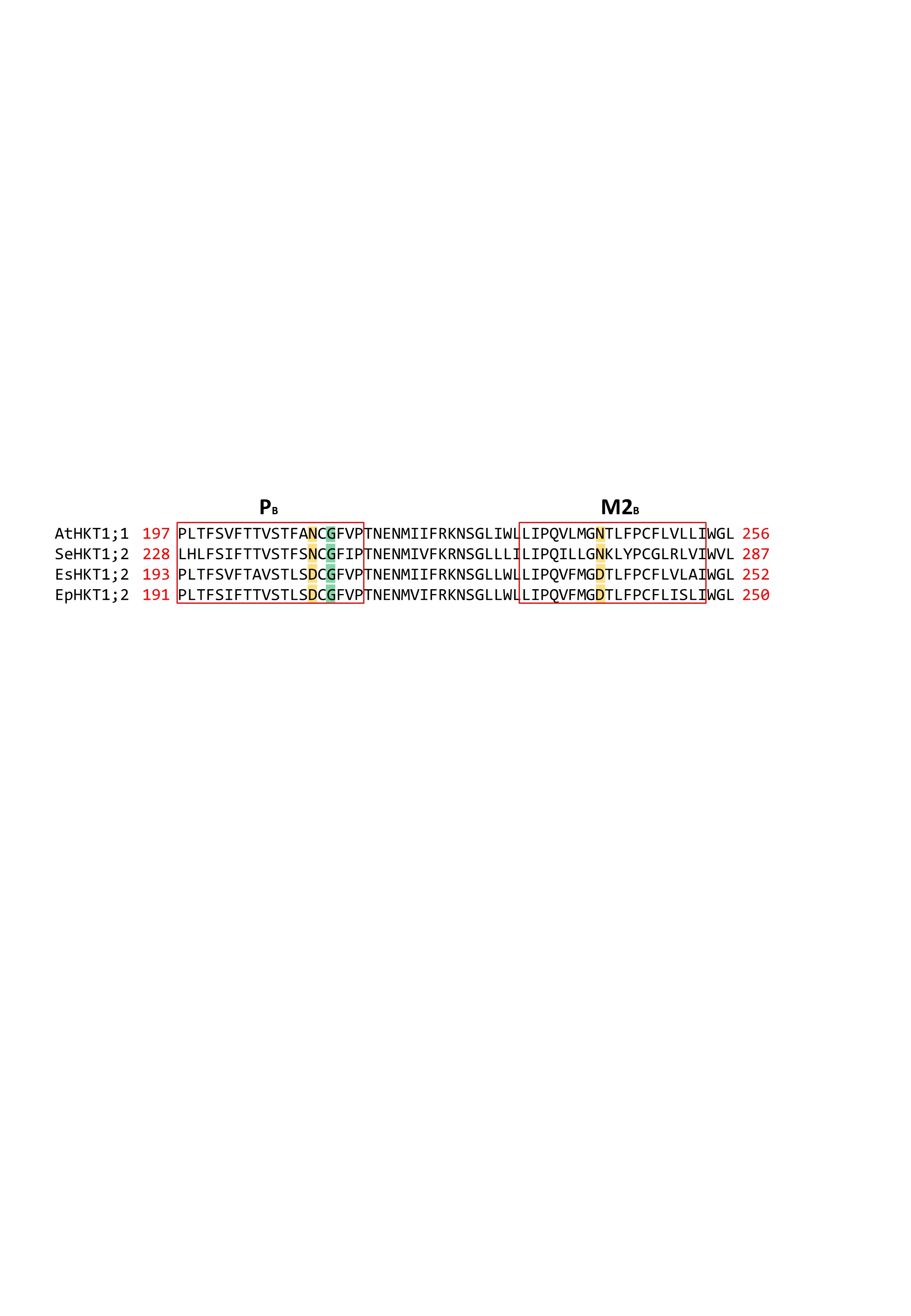

Supplement: Supplementary file 5 [file Image_5.jpeg]
