# Supplementary material for: A novel high-affinity potassium transporter SeHKT1;2 from halophyte Salicornia europaea shows strong selectivity for Na+ rather than K+
Source: Front Plant Sci. 2023 Feb 20;14:1104070. doi: 10.3389/fpls.2023.1104070 (PMC9986455; doi:10.3389/fpls.2023.1104070)
Supplement: Supplementary file 6 [file Table_1.docx]

**Supplemental figure legends**

**Figure S1. Cloning of *SeHKT1;2* gene form *Salicornia europaea*.** **(A)** Evaluation of amplified *SeHKT1;2* gene sequence. **(B)** Multiple sequence alignment and conserved domain analysis of *SeHKT1;2* and *SeHKT1;1* protein.

**Figure S2. Prediction of *SeHKT1;2* protein functional domain and secondary structure. (A)** Graphical representation of the *SeHKT1;2* protein domains. The domains were predicted by SMART data base (<http://smart.embl.de/>) **(B)** Secondary structure of *SeHKT1;2* protein. The secondary structure was predicted by NetSurfP - 3.0 online program (<https://dtu.biolib.com/NetSurfP-3/>).

**Figure S3. 3D modelling of *SeHKT1;2* protein structure. (A)** Predicted 3D structure of *SeHKT1;2* protein. The blue part is more reliable compare to red part. **(B)** Model quality estimated by QMEAN function. **(C)** Global scoring (Z-score) in relation to a set of high-resolution PDB structures.

**Figure S4.** **Functional compression of *SeHKT1;2* in N^+^ sensitive yest strains G19 and AXT3K.** Growth of G19 and AXT3K yeast cells transformed by empty vector (pYSE) control and pYSE-SeHKT1;2. Each transformants on solid arginine phosphate (AP) medium supplemented with various salt concentrations (0, 25, 30, 60, 100, and150 mM of NaCl) were incubated at 30℃ for 3 days. Numbers (1, 10^-1^, 10^-2^, 10^-3^) on the top of each panel indicated serial dilutions of yeast cells placed on the medium.

**Figure S5.** **Sequence comparison** **analysis of HKT homologs**. Amino acid sequences in the second pore loop region (PB) and the adjacent transmembrane domain (M2B) of four HKT proteins were aligned by ClustalW. The conserved Gly residues in the PB region are indicated by green box whereas Asp and Asn residues were highlighted with box.

**Table S1: List of the primers used in this study**

**Table S2: List of HKT proteins used for phylogenetic analysis in this study**
